# Protocol for a process evaluation of a cluster randomized controlled trial of the Learning Club intervention for women's health, and infant's health and development in rural Vietnam

**DOI:** 10.1186/s12913-019-4325-5

**Published:** 2019-07-23

**Authors:** Jane Fisher, Trang Nguyen, Thach Duc Tran, Ha Tran, Tuan Tran, Stanley Luchters, David Hipgrave, Sarah Hanieh, Beverley-Ann Biggs

**Affiliations:** 1 Global and Women’s Health, School of Public Health and Preventive Medicine, Monash University, 553 St Kilda Road, Melbourne, Victoria 3004 Australia; 2 Research and Training Centre for Community Development (RTCCD), Hanoi, Vietnam; 3 Burnet Institute, Melbourne, Australia; 4 International Centre for Reproductive Health, Department of Obstetrics and Gynaecology, Ghent University, Ghent, Belgium; 5 UNICEF, New York, USA; 6 Melbourne School of Population and Global Health, University of Melbourne, Melbourne, Australia; 7 Department of Medicine and Victorian Infectious Diseases Service at the Doherty Institute, University of Melbourne, Melbourne, Australia

**Keywords:** Learning Clubs intervention, Early childhood development, Process evaluation, Vietnam

## Abstract

**Background:**

*Learning Clubs* is a multi-component intervention to address the eight common risk factors for women’s health, and infant’s health and development in resource-constrained settings. We are testing in a cluster randomized controlled trial in rural Vietnam whether this intervention improves cognitive development in children when they are aged two. There are few comprehensive process evaluations of complex interventions to optimise early childhood development. The aim is to conduct a planned process evaluation of the Learning Clubs intervention in Vietnam.

**Methods:**

The evaluation will be conducted alongside the Learning Clubs trial using both qualitative and quantitative methods. Four domains will be included in the evaluation: [1] Context – how contextual factors affect the implementation and outcomes; [2] Implementation – what aspects of the Learning Clubs intervention are actually delivered and how well the intervention is delivered; [3] Mechanism of impact – how the intervention produces changes in the primary and secondary outcomes; and [4] National integration – how the intervention can be scaled up for application nationally. Purposive sampling will be used to recruit project stakeholders from commune, provincial and national levels. Results of the process evaluation will be integrated with those of the outcome and economic evaluations to provide a comprehensive picture of the effectiveness of the Learning Clubs intervention for early childhood development in rural Vietnam.

**Discussion:**

Results of the evaluation will provide evidence about the implementation of the intervention and explanations for any differences in the outcomes between participants in intervention and control conditions. The evaluation will be integrated into each stage of the outcome assessments, but will be implemented by a bilingual team independent of the team implementing the intervention. It will therefore provide evidence which will not be influenced by or influence the intervention and will inform both generalisation to other settings and scalability in Vietnam.

**Trial registration:**

Trial registration number ACTRN12617000442303 on the Australian New Zealand Clinical Trials Registry. Registered 27/03/2017. Prospectively registered.

**Electronic supplementary material:**

The online version of this article (10.1186/s12913-019-4325-5) contains supplementary material, which is available to authorized users.

## Background

Early childhood development, addressed in three Lancet series, is now a global priority [1–3]. More than 40% of children aged under five years living in low- and middle-income countries (LMICs) do not reach their developmental potential. Risks accrue from conception. During pregnancy, women in LMICs experience malnutrition, poverty, gender-based violence, the common mental disorders of depression and anxiety and inadequate access to health care and social protection each of which exerts adverse effects on fetal and infant growth, health and development. Among women in rural Vietnam who are pregnant, 32% experience food insecurity, 20% having a Body Mass Index (BMI) < 18.5, 80% are iodine deficient, 17% have iron deficiency anaemia, 19% experiencing intimate partner violence and one third meet criteria for a common mental disorder [4–7]. Risks interact to influence each domain of development. Children of mothers who have experienced iron deficiency anaemia and antenatal common mental disorders (CMD) have worse cognitive development at the age of six months. Early social and emotional development is worse among children whose mothers experienced postnatal CMD, who had lower parenting confidence and who provided less affectionate care. Father’s behaviours are also relevant, with children whose mothers experienced violence perpetrated by their intimate partners being more likely to be stunted as toddlers. [4, 8–11]

Investment in strategies to promote early childhood development is fundamental to achieving sustainable development. Multi-component, cross-sectoral intervention packages are recommended to enable children to realise their development potential [1, 2, 12].

### Learning clubs: a cluster randomized controlled trial to improve women’s health and infant’s health and development in Vietnam

Informed by our detailed local epidemiological evidence, the Learning Clubs for Women’s Health and Infant Health and Development (Learning Clubs) multi-component, multi-sectoral intervention was developed to improve the physical and mental health of women and the health and development of their infants in rural Vietnam, by addressing multiple risks together [13].

The Learning Clubs intervention is a structured program that combines specific information, learning activities and social support in accessible facilitated community-based groups of women at the same gestational stage, and, after birth, with their babies. The intervention comprises 19 educational modules, delivered in face-to-face groups by locally trained facilitators at a community centre, and one home visit. Unlike existing programs which have aimed to address one or at most two risks, the Learning Clubs intervention is innovative in addressing all identified risks to early childhood development in a comprehensive integrated program. All content is drawn from interventions shown in RCTs in resource-constrained settings to be effective in addressing either maternal nutrition, mental health, parenting capabilities, infant health and development, or gender-based violence and empowerment. These have been adapted to be consistent with current national perinatal programs in Vietnam. Each module comprises information and activities which aim to increase perinatal stage-specific essential knowledge and skills through structured learning opportunities. They have been translated, culturally adapted and field-tested for salience and comprehensibility. Take home print materials, short DVD clips, posters and a structured facilitator’s guide are used for fidelity and to reinforce key messages. The cluster randomised controlled trial of the Learning Clubs intervention will follow women from early pregnancy (baseline survey), and, with their children, to two years after birth (in three follow-up surveys). They will be recruited from 84 communes, half randomly allocated by an independent statistician to each trial arm, in a rural province in Vietnam [13].

### Process evaluation of complex interventions

In general, published accounts of complex interventions report on outcomes, but do not provide details about implementation. These are however essential for generalization of effective interventions to other settings and for taking them to scale in the setting in which they have been tested. Process evaluations seek to describe not only how the intervention “worked”, but also how it was conducted, the underlying mechanisms of change and the effects of context-specific factors. Reviewing 542 articles and reports which include information about intervention implementation processes, Durlak et al. (2008) [14] concluded that in promotion and prevention programs, the outcomes are highly dependent on implementation factors. Similarly, Smith et al. (2004) [15] reviewed 14 school-based anti-bullying programs and found that those which monitored implementation had a mean effect on self-reported bullying and victimization rates up to twice that of those which did not monitor implementation. In some reviews, when taking implementation factors into account the difference in effect sizes was as large as 12-fold [16].

The UK Medical Research Council recommends that a process evaluation of implementation be conducted for all trials of complex intervention. It will help to identify why interventions are effective and how to improve those that are not. It will also provide researchers, program evaluators and policymakers with essential information to assess the possibility of replication and suitability of the intervention in their specific settings, as well as the “contextual factors associated with variation in outcomes” [17]. The Learning Clubs cluster-randomised controlled trial is a world-first evaluation of a complex intervention addressing women’s health and infant’s health and development in a resource-constrained setting. A process evaluation conducted alongside the trial will not only inform policymakers, clinicians and researchers in Vietnam; it will increase the chance of replication of this model in other LMICs.

## Methods

### Aims and objectives

**Fig. 1:**
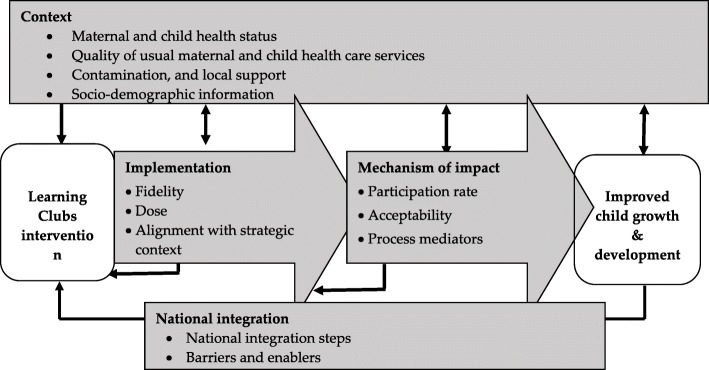
Process evaluation components

The primary aims of the process evaluation are to describe how the intervention works in the resource-constrained setting of rural Vietnam and how to integrate the model into existing national programs. Four domains are monitored to address these aims. The specific content of these domains is presented in the theoretical framework below (see Fig. 1).

- Domain 1 – Context: to explore how contextual factors affect the implementation and outcomes;
- Domain 2 – Implementation: to explore what aspects of the Learning Clubs are actually delivered and how well the Learning Club intervention is delivered;
- Domain 3 – Mechanism of impact: to understand how the Learning Clubs intervention produces changes in the outcomes;
- Domain 4 – National integration: To explore how the Learning Club can be scaled up at the national level

### Learning Club intervention

The Learning Clubs intervention is a community-based structured program which is delivered to women from when they are less than 20 gestational weeks pregnant, to one year after birth.

The cluster randomised controlled trial of the Learning Clubs intervention will follow women from early pregnancy (baseline survey), and, with their children, to two years after birth (in three follow-up surveys). All women recruited from 84 communes (half the communes will be randomly allocated by an independent statistician to each trial arm) will receive the current maternal and child health standard of care. Women in the intervention group will receive the Learning Clubs intervention in addition to the standard of care. Women with cognitive and serious physical disabilities who are not able to implement the intervention practices will be ineligible for the study.

The number of clusters and overall sample size were calculated using the clustersampsi module in Stata, Version 13 (StataCorp LP, College Station, Texas, USA). In order to detect a difference in the primary outcome (Bayley Scales of Infant and Toddler Development cognitive development score < 1 SD at 2 years of age) of 15% in the control arm and 8% in the Learning Club intervention arm (with 80% statistical power and a significance level of 0.05; intra-cluster correlation coefficient [ICC] = 0.03), a total of 1,008 pregnant women from 84 clusters is needed [13].

### Setting

The trial will be conducted in 84 communes (the smallest administrative grouping) in Ha Nam province. It is a typical Northern rural province in Vietnam in terms of culture, health care and education systems. Maternal and child health services are provided by the commune health station in each commune.

### Process evaluation design

The process evaluation will use multiple methods to collect qualitative and quantitative data. Small group discussions, semi-structured interviews, case-studies and independent observations will be used to collect qualitative data. Quantitative data will be sought through self-reported questionnaires and secondary analyses of routinely collected data.

#### Informants and sampling

Purposive sampling methods will be used to recruit informants for the qualitative components of the evaluation. Informants will include stakeholders from commune, provincial and national levels of government, including Health Department officers, project implementation officers, commune health workers, early childhood education workers, Women’s Union representatives, and members of the community. We will select participants to ensure diversity in terms of sector represented, extent of experience with implementing the Learning Clubs, and geographic distribution within Ha Nam province. We will continue to recruit stakeholders until we have reached informational redundancy.

At least 504 women will be recruited from communes assigned to the intervention arm and invited to participate. Attendance lists (women, their partners and other family members) are being kept by facilitators at each Learning Club meeting. These will enable us to calculate participation rates by total number of groups attended and by session topic.

#### Timing of data collection

Data will be collected at several time points. The process evaluation will be conducted alongside the four survey rounds of the outcome evaluation (at baseline, when the women are less than 20 weeks gestation; at Follow-up 1 when they are in late pregnancy; Follow-up 2–1 year after birth, and Follow-up 3–2 years after birth).

### Data collection methods

#### Domain 1 - Context

The aim of this component is to evaluate variation in current maternal and child health programs, quality of usual maternal and child health services, contamination from any other relevant programs and the local support among the 84 communes in the intervention and control arms.

*Current maternal and child health services* in each commune will be assessed through obtaining information about 11 core maternal and child health indicators, including two mortality indicators, one indicator, stunting, among children under five years of age and eight coverage indicators. These are the ones recommended by the World Health Organization (WHO), the United Nations’ International Children's Emergency Fund (UNICEF), Countdown to 2015, and the Health Metrics Network to monitor maternal and child health, and child mortality in the world’s high-burden and low-income countries [18]. Primary data sources for these indicators will be the vital registration and health facility reports of the 84 communes in Ha Nam province in 2018, 2019 and 2020 (see Additional file 1).

#### Quality of usual maternal and child health care services

Quality of existing maternal and child health services will be assessed through indicators in three domains: maternal, newborn and general health. These indicators were proposed by WHO to evaluate the quality of maternal and new born health care provided by primary health facilities. Information about these indicators will facilitate comparison of data in this project with international data [19]. The data source for this component will be mainly routine health facility reports (see Additional file 2).

#### Contamination

A data collection form will be developed and distributed to the Women’s Union, provincial government authorities, commune health stations, key social mass organizations in both control and intervention groups to collect information about any related maternal and child health activities such as training, other interventions, new services and information sources that may affect the outcomes of the Learning Clubs. This form will be distributed to project communes at every survey round and completed forms will be posted to the Research and Training Centre for Community Development (RTCCD) in prepaid reply envelopes. In addition, the team will investigate cases of sharing information between the intervention and control groups.

#### Local support

Frequency and active involvement of local authorities and social organizations in maternal and child health activities will be measured to assess local support at each commune. This information will be gathered by a form developed by the process evaluation team. Information sources are the Women’s Union and their related partners. The support may include access to meeting rooms, computer equipment, the involvement of mass social organizations (Youth Union, Famer’s Union) in advocacy activities, financial support for Learning Club staff, or provision of other necessary equipment. Types of support and frequency of access, activities, or use will be assessed.

#### Other socio-economic characteristics of the commune

In addition information, such as population size, commune health facilities and human resources, number and nature of factories or construction sites located in the commune and neighboring areas, and environmental pollution sources, will be obtained at baseline and during the Learning Clubs intervention. These factors may result in common illnesses among children and mothers and affect their health.

#### Domain 2 - Implementation

This domain will evaluate the implementation aspect of the Learning Club intervention, especially in terms of the quality (fidelity), quantity (dose) and its alignment with the current strategic context in Ha Nam and in Vietnam.

#### Intervention fidelity and dose

There are different definitions of “fidelity” [20–25]. Commonly, it refers to the degree to which an intervention is implemented as planned. In this study, “fidelity” is defined as the quality of the intervention delivered, while “dose” is the quantity of the intervention such as frequency, duration, and coverage [26, 27]. Structurally, the Learning Club intervention content can be divided into two periods: during pregnancy, and when the infants are 0–1 year old. After each period, workshops will be conducted among all facilitators to obtain information about their experience in operating the clubs and in delivering the intervention contents. During these workshops, semi-structured interviews and small group discussions with the club facilitators will be conducted to identify any modification in delivering the intervention, in terms of the content and dose, and to understand reasons for these modifications, if any.

In addition, during the club meetings, a team of provincial trainers will undertake routine supportive supervision and observations of Learning Club meetings. A supervision checklist will be developed for this team to record any variations and report any changes observed in the meetings.

#### Alignment with strategic context

In order to prepare for the scaling up phase, an important element to be considered is the appropriateness of the intervention for the local context. This is defined as the relevance and perceived fit of the intervention in maternal and child health policies and interventions [28]. At Baseline and Follow-up 3, secondary data collection and semi-structured interviews with key stakeholders at national, provincial and commune levels of the education, health and labor sectors and Women’s Union will be held. Information about the strategies, regulations and vision in terms of maternal and child care in the local communes will be sought. The purpose of this component is to identify relevant government strategies, which may enable the integration of the intervention into in national programs. It is estimated that around 10 interviews will be undertaken at each survey round.

#### Domain 3 - Mechanisms of impact

#### Participation rate

At each Learning Club’s meeting, a participation form will be used by the facilitators to record the number of people attending the session. The form and (with participant consent) a photo of the meeting will be sent to the project officers. Weekly and monthly reports will be prepared by the project officers to establish participation rates. These reports will be submitted to the project coordinator, the process evaluation team and the research advisory group.

#### Acceptability

Small group discussions with Learning Club participants will be held at Follow up 1 (F1), Follow up 2 (F2) and Follow up 3 (F3) to explore their experiences of and views about attending Learning Clubs sessions; advantages, difficulties and unexpected outcomes of participation will also be sought. Participants in these discussions will be recruited using maximum variation purposive sampling. This type of sampling design allows the recruitment of outliers whose experiences are diverse to maximize the variety of perspectives among people contributing data. Therefore, Learning Club participants who are more and less able to apply the Club content in self-care and care of their infants will be included to explore underlying factors that might influence the outcomes.

#### Process mediators

Process mediators, including changes in facilitators’ knowledge about early childhood development, and skills and confidence in Club operations; participants’ changes in knowledge and skills in providing care for their child, sharing of paid and unpaid work with an intimate partner and quality of family relationships; and support from family members to the club’s mothers will be collected at F1, F2 and F3 through small group discussions and a self-administered questionnaire.

#### Domain 4 - National integration

#### National integration steps

The process evaluation officer will work closely with the project implementation team to document all steps taken to ensure that the Learning Club content is harmonized with existing national programs. Information about steps taken in the preparation, implementation and post-intervention phases, to engage local authorities, relevant stakeholders and policymakers in the project, will be gathered using a project diary. This component will provide descriptions of purposeful steps that the project team has conducted to inform policy makers and advocate for the intervention.

#### Barriers and enablers

Semi-structured interviews and small group discussions will be organized to identify the barriers and enablers for national integration of the Learning Clubs, from each stakeholder’s perspective. Key representatives from government agencies (Women’s Union, health, education and labor sectors) at national, provincial and commune levels will be invited to participate. The views of local and international non-government and United Nations agencies, including the World Health Organization, UNICEF, and the United Nations Population Fund (UNFPA), about the intervention will be also be sought. It is estimated that about 25 interviews will be conducted in total by the process evaluation team.

### Data management and analysis

Consent will be sought from participants for all small group discussions and semi-structured interviews to be audio-recorded. Data will be transcribed by experienced researchers in Vietnamese and then translated into English so that investigators in this bi-cultural team can co-contribute to analyses. Data will be uploaded to a secured cloud-based storage system in Monash University, Australia. Only authorized researchers will have access to the data.

Transcripts will be entered into the NVivo version 11.0 and data will be analyzed thematically. Frameworks for coding of data will be established and revised as new themes emerge. Themes reflecting the context, implementation, and mechanism of impacts will be used in the interpretation of the impacts of the Learning Clubs on ultimate and intermediate outcomes. They may also be used to inform post-hoc secondary analysis of the outcomes, for instance, to examine whether contamination by other relevant projects may diminish the impacts of the Learning Clubs on infants’ cognitive development.

#### Domain 1 – Context

Data collected from Domain 1 will yield a comprehensive map and description of the context and influencing factors of maternal and child health in the 84 participating communes. These will enable differences in outcomes between control and intervention arms to be understood in this context. The four key contextual factors to be considered will be maternal and child health status, quality of maternal and child health services, local support and social and economic characteristics. The indicators of the maternal and child health status, the quality of related usual care services will be analyzed and compared to other settings using the guidelines developed by the World Health Organization and its partners [18, 19].

#### Domain 2 – Implementation

The fidelity and dose of the intervention will be categorized into high, medium and low levels of fidelity and dose. This provides evidence of the variation in delivery of the intervention, especially in terms of what aspects of the intervention have been delivered, how well they have been delivered, and reasons for any modifications made. These modifications will reflect what the Learning Club facilitators needed to do to fit the intervention to the real situations in their communes. It will help the project team to revise the content and structure of the intervention to meet the capacity of the facilitators in rural areas. In addition, the alignment with the strategic context will enable mapping and adjusting the Learning Club intervention to existing policies and regulations related to maternal and child health in Ha Nam. It will enable the project team and policymakers to identify relevant national programs that the project may contribute to.

#### Domain 3 – Mechanism of impact

Domain 2 provides information on the acceptability to the facilitators of operating the Learning Clubs, and Domain 3 ascertains the accessibility of and acceptability to the target audience. The participation rate reflects the involvement of mothers, their intimate partners and other family members in the Club meetings. Qualitative information from the small-group discussions will inform revisions of the intervention, in terms of content and format, if required, to meet participants’ needs more effectively. It will inform the dissemination strategy for the final scaling-up phase, if this is indicated. Moreover, information about the process mediators will contribute to ascertaining whether the underlying causal assumptions of the intervention do or do not lead to differences in the outcomes between trial arms.

Case studies will be developed to explain the causal relationship between the input and the outcome conditions. The qualitative data will inform revisions, if necessary, to the theory of change.

#### Domain 4 – National integration

This component provides a detailed description of the integration procedure into existing relevant national programs. It will also provide the structure and rationale for each step of the implementation and evaluation of the intervention. This evidence for policy makers in Vietnam will enable the production of guidelines for integrating community-based initiatives into the government health and social protection systems.

### Integration of process evaluation and outcomes findings

In addition to assessing the primary and secondary outcomes, this process evaluation is one of the two evaluations to be conducted alongside the main cluster randomized trial, the other is an economic evaluation. There is still debate among researchers about how to integrate process evaluation data into outcome and cost-effectiveness evaluations. In this project, after the final data collection is completed, the process evaluation data will be combined with the other evaluations to enable a comprehensive picture of the effectiveness of this community-based intervention to be prepared. Quantitative process data will be integrated into the analysis of outcomes and cost-effetiveness using regression models and path analysis. Qualitative components will provide insights to explain the causal pathways of changes in the outcomes.

### Blinding

The process evaluation will be conducted independently by two experienced health researchers who are bilingual in English and Vietnamese. These researchers will be able to conduct interviews and analyze data in both languages and will not be involved in implementing any project activities. As one of the aims of the Learning Clubs project is to establish whether the Learning Clubs intervention is effective under real-world conditions, the process evaluation team will not provide feedback to the Learning Club project staff, project stakeholders, and project advisory group or exchange relevant information that may result in changes to the project. Information obtained from the process evaluation will be taken into account in the final analysis and interpretation of the results of the main trial and will be published separately.

### Dissemination strategy

The data will be used to write peer-reviewed publications, workshop presentations and technical reports. In addition, it will be included in the policy brief to advocate for integrating the intervention nationally. No personal information will be provided in any documents released from the project.

## Discussion

This paper describes the protocol of a process evaluation conducted alongside a cluster randomized trial to promote early childhood development in a rural province in Vietnam. Outcomes of the process evaluation will provide insights into the implementation process of the project and explain the causal pathways between the Learning Club intervention and any outcomes achieved. It will also provide evidence about the barriers and enablers for implementation of a community-based intervention in in a lower-middle income country. Lessons learned from the study could be helpful for the scaling-up phase in Vietnam as well as the implementation or adaptation of the intervention in other LMICs.

The process evaluation is conducted alongside the trial and is integrated into the four surveys of the main trial. It allows the evaluation team to reach stakeholders in the study without placing too much burden on them. In addition, it also utilizes resources of the main trial in terms of traveling to the region and local infrastructure.

The process evaluation is implemented by an independent bilingual, bi-cultural team which can work effectively and communicate well with both the Vietnamese partners and the English-speaking members of the research team. They will be independent and will not communicate with the implementation team. To ensure the trial is being conducted objectively and as close to reality as possible any problems identified will not be shared during the implementation process.

Nevertheless, we acknowledge some limitations. The process evaluation will be undertaken alongside the four survey rounds of the main trial, it may thus introduce some confounding factors. Participating in semi-structured interviews or focus group discussions, Learning Clubs participants, facilitators and stakeholders may share and form some perceptions about problems they may encounter during the Club's meetings; changes in the way they participate or facilitate the Club's sessions may thus be made and these may result in changes to the main outcomes of the trial.

The process evaluation aims to supplement the outcome and economic evaluations. It also explores the method to scale up the Learning Clubs at the national level by understanding stakeholders’ perspectives about actions for improving early childhood development in Vietnam. It will provide important local information about implementation of a community-based, low-cost intervention in a resource-constrained setting, to improve the development of young children and respond to the needs of their caregivers. It will also be of significant importance to the replication of the program in other LMICs.

## Additional files

Additional file 1: Core indicators of women’s and children’s health (DOCX 18 kb)

Additional file 2: Indicators for assessing quality of maternal and newborn health services (DOCX 15 kb)

## Data Availability

At completion of the trial and following publication of all outcomes, de-identified data will be available through a secure portal at Monash University, which can be accessed with the permission of the investigators and will require a formal agreement for use.

## Abbreviations

BMI: Body Mass Index
CMD: Common mental disorders
F1: Follow-up 1
F2: Follow-up 2
F3: Follow-up 3
LMICs: Low and Middle Income Countries
RCT: Randomized Controlled Trial
RTCCD: Research and Training Center for Community Development
SMD: Severe mental disorders
UNICEF: United Nations’ International Children's Emergency Fund
UNFPA: United Nations Fund for Population Activities
WHO: World Health Organization
