# Supplementary material for: Protocol for a process evaluation of a cluster randomized controlled trial of the Learning Club intervention for women's health, and infant's health and development in rural Vietnam
Source: BMC Health Serv Res. 2019 Jul 23;19:511. doi: 10.1186/s12913-019-4325-5 (PMC6651982; doi:10.1186/s12913-019-4325-5)
Supplement: Supplementary file 1 — Core indicators of women’s and children’s health (DOCX 18 kb) [file 12913_2019_4325_MOESM1_ESM.docx]

**Additional File 1: Core indicators of women's and children's health**

| **No** | **Indicators** | **Explanation** | **2018** | **2019** | **2020** |
| --- | --- | --- | --- | --- | --- |
| 1 | **Maternal mortality ratio** |  |  |  |  |
| 2 | **Under-five child mortality, with the proportion of newborn deaths** | The ratio of the number of recorded (or estimated) maternal deaths during a given time period per 100 000 live births during the same time period (the number of maternal deaths in a population divided by the number of live births – depicting the risk of maternal death relative to the number of live births).  A maternal death refers to a woman’s death from any cause related to or aggravated by pregnancy or its management (excluding accidental or incidental causes) during pregnancy and childbirth or within 42 days of termination of pregnancy, irrespective of the duration and site of the pregnancy. |  |  |  |
| 3 | **Children under five who are stunted** | Percentage of children under five who are stunted  ***Numerator***: Number of children under five years of age whose length-for-age or height-for age is below minus two standard deviations from the median of the World Health Organization (WHO) Child Growth Standards  ***Denominato***r: Number of children under five years of age with a valid length or height measurement |  |  |  |
| 4 | **Proportion of demand for family planning satisfied (met need for contraception)** | Percentage of women of reproductive age (15-49 years or age), either married or in a union, who have their need for family planning satisfied. This indicator is determined by the current levels of contraceptive use and the unmet need for family planning.  ***Numerator***: The Contraceptive Prevalence Rate (CPR) is the percentage of women of reproductive age (15-49 years old) who are married or in a union and who are currently using, or whose sexual partner is currently using, at least one contraceptive method, regardless of the method used (modern or traditional).  ***Denominator***: Total demand for family planning is defined as the sum of the CPR (as defined above) and the unmet need for family planning. Unmet need for family planning is the proportion of women of reproductive age (15-49 years old) either married or in a consensual union, who are fecund and sexually active but who are not using any method of contraception (modern or traditional), and report not wanting any more children or wanting to delay the birth of their next child for at least two years. Included are:   - all pregnant women (married or in a consensual union) whose pregnancies were unwanted or mistimed at the time of conception; - all postpartum amenorrhoeic women (married or in consensual union) who are not using family planning and whose last birth was unwanted or mistimed; - all fecund women (married or in consensual union) who are neither pregnant nor postpartum amenorrhoeic, and who either do not want any more children (want to limit family size), or who wish to postpone the birth of a child for at least two years or do not know when or if they want another child (want to space births), but are not using any contraceptive method. |  |  |  |
| 5 | **Antenatal care coverage (at least four times during pregnancy)** | Percentage of women attended at least four times during pregnancy by any provider (skilled or unskilled) for reasons related to the pregnancy.  ***Numerator***: Number of women attended at least four times during pregnancy by any provider (skilled or unskilled) for reasons related to the pregnancy in the *x* years prior to the survey.  ***Denominator***: Total number of women who had a live birth in the same time period |  |  |  |
| 6 | **Antiretroviral (ARV) prophylaxis among HIV positive pregnant women to prevent HIV transmission and antiretroviral therapy for [pregnant] women who are treatment-eligible** | 1. Percentage of HIV-infected pregnant women provided with antiretroviral drugs to reduce the risk of mother-to-child transmission during pregnancy and delivery.   ***Numerator***: Number of HIV-infected pregnant women who received antiretroviral drugs during the past 12 months to reduce mother-to-child transmission of HIV.   - ARV therapy for HIV-infected pregnant women eligible for life-long treatment - Maternal triple ARV prophylaxis. This includes the following azidothymidine/also called zidovudine (AZT)-based regimens when all three drugs are started simultaneously: - AZT + 3TC + LPV-r - AZT + 3TC + ABC - AZT + 3TC + EFV - Maternal AZT. This includes women who receive only AZT starting at 14 weeks and those that receive other ARVs (such as 3TC and NVP10) at labour and delivery, or postpartum (as a “tail”). A regimen with AZT as the primary prophylactic agent regardless of the duration and receipt of other drugs should be included in this category. - Single-dose nevirapine only (SDNVP).   ***Denominator***: Estimated number of pregnant HIV-infected women within the past 12 months.   1. Percentage of HIV-infected (pregnant) women who are treatment eligible provided with antiretroviral therapy   ***Numerator***: ARV therapy for HIV-infected pregnant women eligible for life-long treatment.  ***Denominator***: Estimated number of HIV-infected pregnant women eligible for ART. |  |  |  |
| 7 | **Skilled attendant at birth** | Percentage of live births attended by skilled health personnel. Skilled health personnel have the required skills to provide life-saving obstetric care, including giving the necessary supervision, care and advice to women during pregnancy, labour and the post-partum period; conduct deliveries on their own; and care for newborns. Traditional birth attendants, even if they receive a short training course, are not included.  ***Numerator***: The number of live births to women ages 15-49 in the *x* years prior to the survey attended during delivery by a skilled health personnel (doctor, nurse, midwife, or auxiliary midwife)  ***Denominator***: Total number of live births to women ages 15-49 in the *x* years prior to the survey |  |  |  |
| 8 | **Postnatal care for mothers and babies within two days of birth** | Percentage of mothers and babies who received postnatal care within two days of childbirth  ***Numerator***:   - Number of women who received postnatal care within two days of childbirth (regardless of place of delivery) - Number of babies who received postnatal care within two days of childbirth (regardless of place of birth)   ***Denominator***:   - Total number of women ages 15-49 years with a last live birth in the *x* years prior to the survey (regardless of place of delivery) - Total number of most recently born babies in the *x* years prior to the survey (regardless of place of birth) |  |  |  |
| 9 | **Exclusive breastfeeding for six months (0–5 months)** | Percentage of infants ages zero to five months who are exclusively breastfed  ***Numerator***: Number of infants zero to five months who are exclusively breastfed  ***Denominator***: Total number of infants zero to five months surveyed |  |  |  |
| 10 | **Three doses of combined diphtheria-tetanus pertussis**  **(DTP3) immunization coverage (12–23 months)** | Percentage of infants who received three doses of diphtheria-tetanus-pertussis vaccine (DTP3)  ***Numerator***: Number of surviving infants (under 12 months of age) who received three doses of diphtheria-tetanus-pertussis vaccine (DTP3)  ***Denominator***: Number of surviving infants (under 12 months of age) in the reference year |  |  |  |
| 11 | **Antibiotic treatment for suspected pneumonia** | Percentage of children ages 0-59 months with suspected pneumonia receiving Antibiotics  ***Numerator***: Number of children ages 0-59 months with suspected pneumonia in the two weeks prior to the survey receiving antibiotics  ***Denominator***: Total number of children ages 0-59 months with suspected pneumonia in the two weeks prior to the survey |  |  |  |
