# Supplementary material for: Protocol for a process evaluation of a cluster randomized controlled trial of the Learning Club intervention for women's health, and infant's health and development in rural Vietnam
Source: BMC Health Serv Res. 2019 Jul 23;19:511. doi: 10.1186/s12913-019-4325-5 (PMC6651982; doi:10.1186/s12913-019-4325-5)
Supplement: Supplementary file 2 — Indicators for assessing quality of maternal and newborn health services (DOCX 15 kb) [file 12913_2019_4325_MOESM2_ESM.docx]

**Additional File 2: Indicators for assessing quality of maternal and newborn health services**

| **No** | **Indicators** | **2018** | **2019** | **2020** |
| --- | --- | --- | --- | --- |
|  | ***Maternal health*** | | | |
| M1 | Proportion of antenatal care visits at which blood pressure was measured |  |  |  |
| M2 | Proportion of women with severe pre-eclampsia or eclampsia treated with magnesium sulphate injection |  |  |  |
| M3 | Proportion of women receiving oxytocin within 1 min of birth of infant |  |  |  |
| M4 | Proportion of women with prolonged labour |  |  |  |
| M5 | Intrapartum stillbirth rate |  |  |  |
| M6 | Proportion of women with severe systemic infection or sepsis in postnatal period, including  Readmissions |  |  |  |
|  | ***Newborn health*** | | | |
| N1 | Proportion of health facilities with functional bags and masks (two neonatal mask sizes) in the delivery areas of maternity services |  |  |  |
| N2 | Proportion of newborns who received all four elements of essential care:   - immediate and thorough drying - immediate skin-to-skin contact - delayed cord clamping - initiation of breastfeeding in the first hour |  |  |  |
| N3 | Proportion of health facilities in which kangaroo mother care is operational, by level of facility |  |  |  |
| N4 | Facility neonatal mortality rate disaggregated by birth weight   - > 4000 g, - 2500–3999 g, - 2000–2499 g, - 1500–1999 g, - < 1500 g |  |  |  |
| N5 | Proportion of health facilities offering maternity services certified by the Baby-friendly Hospital Initiative and recertification no later than 2 years afterwards |  |  |  |
|  | ***General indicators*** | | | |
| G1 | Proportion of health facilities that have stock-outs of essential lifesaving medicines for mothers and newborns in a specified period |  |  |  |
| G2 | Proportion of maternal and perinatal and child deaths occurring in a facility that were reviewed |  |  |  |
| G3 | Proportion of health facilities with soap and running water or alcohol-based rub available in labour, childbirth, neonatal and paediatric wards |  |  |  |
| G4 | Proportion of health facilities with safe, uninterrupted oxygen supply in childbirth, neonatal  and paediatric wards |  |  |  |
